# Heat and drought impact on carbon exchange in an age-sequence of temperate pine forests

**DOI:** 10.1186/s13717-021-00349-7

**Published:** 2022-01-25

**Authors:** M. Altaf Arain, Bing Xu, Jason J. Brodeur, Myroslava Khomik, Matthias Peichl, Eric Beamesderfer, Natalia Restrepo-Couple, Robin Thorne

**Affiliations:** 1 School of Earth, Environment and Society and McMaster Centre for Climate Change, McMaster University, 1280 Main Street West, Hamilton, ON L8S 4K1 Canada; 2 Department of Biological Sciences, University of Calgary, Alberta, Canada; 3 Department of Geography and Environmental Management, University of Waterloo, Waterloo, ON Canada; 4 Department of Forest Ecology and Management, Swedish University of Agricultural Sciences, Umeå, Sweden; 5 School of Informatics, Computing, and Cyber Systems, Northern Arizona University, Flagstaff, AZ USA; 6 Department of Ecology and Evolutionary Biology, University of Arizona, Tucson, AZ USA; 7 Department of Geography, Wilfrid Laurier University, Waterloo, ON Canada

**Keywords:** Carbon fluxes, Net ecosystem productivity, Ecosystem respiration, Extreme weather events, Drought, Temperate forest, White pine, Eddy covariance

## Abstract

**Background:**

Most North American temperate forests are plantation or regrowth forests, which are actively managed. These forests are in different stages of their growth cycles and their ability to sequester atmospheric carbon is affected by extreme weather events. In this study, the impact of heat and drought events on carbon sequestration in an age-sequence (80, 45, and 17 years as of 2019) of eastern white pine (*Pinus strobus* L.) forests in southern Ontario, Canada was examined using eddy covariance flux measurements from 2003 to 2019.

**Results:**

Over the 17-year study period, the mean annual values of net ecosystem productivity (NEP) were 180 ± 96, 538 ± 177 and 64 ± 165 g C m^–2^ yr^–1^ in the 80-, 45- and 17-year-old stands, respectively, with the highest annual carbon sequestration rate observed in the 45-year-old stand. We found that air temperature (Ta) was the dominant control on NEP in all three different-aged stands and drought, which was a limiting factor for both gross ecosystem productivity (GEP) and ecosystems respiration (RE), had a smaller impact on NEP. However, the simultaneous occurrence of heat and drought events during the early growing seasons or over the consecutive years had a significant negative impact on annual NEP in all three forests. We observed a similar trend of NEP decline in all three stands over three consecutive years that experienced extreme weather events, with 2016 being a hot and dry, 2017 being a dry, and 2018 being a hot year. The youngest stand became a net source of carbon for all three of these years and the oldest stand became a small source of carbon for the first time in 2018 since observations started in 2003. However, in 2019, all three stands reverted to annual net carbon sinks.

**Conclusions:**

Our study results indicate that the timing, frequency and concurrent or consecutive occurrence of extreme weather events may have significant implications for carbon sequestration in temperate conifer forests in Eastern North America. This study is one of few globally available to provide long-term observational data on carbon exchanges in different-aged temperate plantation forests. It highlights interannual variability in carbon fluxes and enhances our understanding of the responses of these forest ecosystems to extreme weather events. Study results will help in developing climate resilient and sustainable forestry practices to offset atmospheric greenhouse gas emissions and improving simulation of carbon exchange processes in terrestrial ecosystem models.

## Background

North American forests are a critical component of the global carbon cycle because they offset a large portion of global fossil fuel carbon dioxide (CO_2_) emissions (Houghton et al. 1999; Pan et al. 2011; Williams et al. 2012). The temperate forests in Eastern North America are dominated by different-aged stands that have been either planted or have naturally regrown since the last major harvests in the nineteenth and twentieth century (OMNRF 1986). Many of these forests have been actively managed to enhance their growth and timber production (Gilliam 2016). However, variations in environmental conditions may have a profound impact on the growth and carbon uptake of these managed forests. While the warming-induced extended growing season and CO_2_ fertilization effects are expected to increase carbon assimilation (Randerson et al. 1999; Nemani et al. 2003; Schwartz et al. 2006), extreme events such as heat and drought may negatively affect net carbon uptake of these forests by limiting photosynthesis and/or increasing soil respiration and litter decomposition, or even altering the forest structure (Krishnan et al. 2006; Allard et al. 2008; Holst et al. 2008; Schwalm et al. 2012; von Buttlar et al. 2018; Xu et al. 2020; Beamesderfer et al. 2020).

Past observations and future climate predictions suggest an increasing frequency and severity of extreme events, such as heat and drought, in mid-latitude regions (Meehl et al. 2007; IPCC 2014, 2018). Some studies suggest that the occurrence of heat and drought events may be shifted into both the spring and autumn shoulder seasons or they may occur simultaneously (Monson et al. 2005; Piao et al. 2008; Hanson and Weltzin 2000; Schwarz et al. 2004; Sippel et al. 2016). The change of severity and timing of climate events may have a profound impact on the carbon cycle in temperate forests (Zscheischle et al. 2014; van Gorsel et al. 2016; Hogg et al. 2017; Wu et al. 2017; Fernández-Martínez et al. 2020). For example, in Europe, a severe summer heatwave and drought caused forest ecosystems to lose large amount of carbon in the summer of 2003 (Ciais et al. 2005; Bréda et al. 2006; Granier et al. 2007). The heatwave of 2018 affected a large part of Europe and caused lower forest productivity than that in 2003 (Buras et al. 2020). In North America, a severe summer drought in 2012, that coincided with warm spring temperatures, significantly impacted carbon exchanges in the region (Wolf et al. 2016). Although increased carbon uptake due to warmer spring temperatures in 2012 helped in compensating the reduction in carbon sequestration due to summer drought, rapid depletion of soil water content also enhanced summer heating through land surface–atmosphere feedbacks (Wolf et al. 2016; Pan and Schimel 2016).

The sensitivity of carbon sequestration to climate constraints and extreme events may vary with stand age because of changes in structural and physiological characteristics, such as leaf area, woody biomass allocation, tree hydraulic conductance and non-structural carbon pools (Niinemets 2010; Peichl et al. 2010a). Mature forests with larger rooting systems have access to deeper water stores, which results in less severe impacts on their carbon uptake and water stress due to heat and/or drought events as compared to younger stands. The canopies of mature stands are often exposed to more sunlight due to their structure—increased tree height and size as compared to younger stands, resulting in higher temperatures and atmospheric humidity deficit within the canopy, which may enhance evaporative demand, leading to stomatal closure and a reduction in photosynthetic rates (Niinemets 2010). These responses indicate considerable complexities associated with the response of different-aged forests to environmental constraints and climatic extreme events. In the literature, many studies have focused on carbon exchanges in different-aged or rotations (the period between planting and harvest) of temperate forests (Peichl et al. 2010a; Law et al. 2003; Clark et al. 2004; Humphreys et al. 2006; Krishnan et al. 2009; Amiro et al. 2010; Goulden et al. 2011; Baldocchi 2019; Xu et al. 2020). However, there is a need for studies that can improve our understanding of how different-aged managed forests respond to multiple environmental drivers, such as concurrent heat and drought events (Williams et al. 2013; Ruehr et al. 2012; Allen et al. 2010; Reichstein et al. 2013; Frank et al. 2015).

The main objectives of this study were to (i) examine the impacts of heat and drought events on carbon exchanges in an age-sequence (80, 45, and 17 years as of 2019) of eastern white pine forests in Eastern North America using eddy covariance flux measurements from 2003 to 2019 and (ii) determine how these different-aged plantation forests may respond to the concurrent or consecutive occurrence of these extreme weather events. The unique, long-term (17 years) data set provided an opportunity to explore the impacts of extreme events occurring over nearly two decades in these stands. We hypothesized that (i) the net ecosystem productivity (NEP) of young stands will be more sensitive to heat and drought stress as compared to older stands, (ii) drought stress will have a more significant impact on NEP when it occurs concurrently with heat stress, and (iii) the consecutive occurrences of heat and/or drought events over multiple years will cause higher reduction in NEP in the younger stands.

## Methods

### Site descriptions

The study sites consist of three eastern white pine forests (*Pinus strobus* L.) planted in 1939, 1974 and 2002 as monoculture stands north of Lake Erie in southern Ontario, Canada. These stands were 80, 45 and 17 years old as of 2019 and are located within 20 km of each other at approximately the same latitude (Table 1). In Eastern North America, white pine is an important native forest species because it is adapted to nutrient poor, well-drained sandy soils and dry environmental conditions. It can grow up to 40 m height and has a lifespan of 380 to 425 years (Thompson et al. 2006). In the pre-European settlement landscape of Eastern North America, old-growth white pine stands covered 50% of the area in the Great Lakes—St. Lawrence Forest region and 30% of the southern boreal forest region (Quinby 1993). During recent decades, white pine has been planted on degraded or disturbed (fire or clearing) lands as the first woody species to eventually facilitate in the establishment of native deciduous forest species through succession (Parker et al. 2001; Arain and Restrepo-Coupe 2005).

**Table 1:** Site characteristics of the 80-year-old, 45-year-old and 17-year-old forests of the Turkey Point Observatory

Site	80-year-old	45-year-old	17-year-old
Stand age (start/end)	64/80	29/45	1/17
Tower location	42.7102 − 80.3574	42.7074 − 80.3485	42.6617 − 80.5599
Elevation (m)	184	184	265
Plantation year	1939	1974	2002
Average canopy height (m)	23.4 (22.9)	16.2	6.85
Tree density (trees ha^–1^)	321 (413)	1583	1567
Leaf area index, LAI (m^2^ m^–2^)	5.3 (8.5)	6.6	6.4
Mean diameter at 1.3 m height, DBH (cm)	38.99 (37.2)	17.90	15.76
Stand basal area (m^2^ ha^–1^)	36.0 (40.9)	40.0	31.8
Above ground biomass (t C ha^–1^)	129.62 (143.8)	86.68	44.67
Soil texture	Fine sand	Fine sand	Fine sand
Proportion of sand (%)	98	85	93
Bulk density (g cm^−3^)	1.357	1.376	1.443

Biometric data were measured in 2012, except numbers in brackets which indicate pre-thinning (2011) data

Soil data are from Khalid 2016

Our forest sites are part of the Turkey Point Observatory and have been associated with the Global Water Futures (GWF) program, former Fluxnet-Canada/Canadian Carbon Program, AmeriFlux and global Fluxnet networks, where they are also known as CA-TP1 (Arain 2018a), CA-TP3 (Arain 2018b) and CA-TP4 (Arain 2018c) sites, respectively. They have also been referred to as TP02, TP74, and TP39 in some studies based on the year forests were planted. The 80-year-old and 45-year-old forests were planted with white pine seedlings on cleared oak-savanna lands, while the 17-year-old forest was planted on a former agricultural land that was left as fallow for several years prior to plantation. Tree species at the 80-year-old site include 82% white pine, 11% balsam fir (*Abies balsamifera* L. Mill) and 7% native Carolinian species—oak (*Quercus velutina* L.*, Q. alba* L.), red maple (*Acer rubrum* L.), wild black cherry (*Prunus serotina* Ehrh.), and white birch (*Betula papyrifera*). The understory at this site consists of young white pines ranging from 0.5 to 6 m tall, black oak, balsam fir, and black cherry. Ground vegetation includes bracken fern (*Pteridium aquilinum*), moss (*Polytrichum *spp.), blackberry (*Rubus *spp.)*,* poison ivy *(Rhus radicans*), and Canada mayflower **(***Maianthenum canadense*). In 1983, thinning was performed at the 80-year-old site; in which 104.76 m^3^ ha^–1^ wood volume was removed from 38.6 ha area (Ontario Ministry of Natural Resources and Forestry records). In the winter of 2012, thinning was again conducted in the 80-year-old site during which one third of the trees were commercially harvested, reducing the stand density from 413 trees ha^–1^ to 321 ± 111 trees ha^–1^ while also reducing the basal area by 13%. Species composition at the 45-year-old stand is 94% white pine, mixed with 5% jack pine (*Pinus banksiana*) and 1% oak. Ground vegetation at the 45-year-old site is dominated by the bryophyte species. The 17-year-old site consists entirely of white pine species. Due to high tree-canopy shading at this site, the understory is limited to very few bryophytes and grasses.

The topography at all three sites is predominantly flat with occasional (0.5–3.0%) undulating slopes. The soils are composed of ~ 98% sand and are classified in the Canadian System of Soil Classification as lacustrine-derived Brunisolic grey–brown luvisols (Presant and Acton 1984). The soils are well-drained and have a low water holding capacity (Table 1; McLaren et al. 2008; Khalid 2016). The water table depth at the 80- and 45-year-old sites is approximately 4–5 m in the winter–spring and about 6–7 m in the summer–autumn. The 17-year-old site is in proximity to Big Creek, where surface water level in the creek is more than 5–6 m below the elevation of the adjacent forest. Further site details are given in Table 1 and in Arain and Restrepo-Coupé (2005), Arain (2018a, [Bibr CR7][Bibr CR5]c), Peichl and Arain (2006), Peichl et al. (2010a) and Beamesderfer et al. (2020). The climate in the region is warm humid continental with a 30-year (1980–2010) mean annual temperature of 8.0 °C and mean annual precipitation of 1036 mm, with approximately 13% falls as snow, based on data records from the Environment and Climate Change Canada weather station at Delhi, Ontario, about 19 to 22 km north-northwest of our sites.

### Flux and meteorological measurements

Half-hourly fluxes of momentum, energy, water vapor, and carbon dioxide (CO_2_) (F_c_) were measured from 2003 to 2019 at the 80-year-old site using a closed-path eddy covariance (EC) system. A single roving open-path EC system was used from 2003 to 2007 at the two other sites, before it was replaced by closed-path EC systems installed at the 45- and 17-year-old sites in January and May 2008, respectively. Each EC setup follows detailed protocols developed by the Fluxnet-Canada Research Network (FCRN). The closed-path EC systems consisted of a sonic anemometer (model CSAT3, Campbell Scientific Inc. (CSI)), an infrared gas analyzer (IRGA, model LI-7000, LI-COR Inc.), a climate control box with a heated sampling tube (4 m) and a desktop PC housed in a trailer/hut (Arain and Restrepo-Coupé 2005). The open-path EC system consisted of a sonic anemometer (model CSAT3, CSI), an IRGA (model LI-7500, LI-COR Inc.), a temperature/relative humidity sensor (model HMP45C, CSI) and a data logger (model CR5000, CSI) to control system operations and store flux data. The open-path system was rotated on biweekly to monthly time intervals among three young forest sites from 2003 to 2007. However, one of these stands (planted in 1989) was decommissioned in 2008. Therefore, only 4 months of data per year was available at the 45- and 17-year-old sites from 2003 to 2007, introducing relatively larger level of uncertainties and errors in the gap-filled flux data. EC sensors were installed at 28 m height on a scaffolding tower at the 80-year-old site, which was increased to 34 m in May 2016. EC sensors were installed at 16 m height on top of a triangular tower at the 45-year-old site, which was replaced with a scaffolding tower, where sensors moved to 20 m height in January 2008 due to increases in tree heights. At the 17-year-old site, flux measurements were initially made at about 2 m height using a triangular tower, which was replaced with a scaffolding tower in July 2014. At this site, the height of EC sensors was gradually increased to maintain approximately 2 m distance above the tree tops as the stand grew. In all three EC systems, air was sampled at 20 Hz and IRGAs were calibrated on bi-weekly to monthly intervals.

CO_2_ storage in the air column below the EC sensors at the 80-year-old site was calculated using CO_2_ concentrations measured from the IRGAs at the top (28 or 34 m) and mid-canopy (14 m) heights (model LI-820/Li-800, LI-COR Inc.). Similarly, storage fluxes at the 45-year-old site were calculated using CO_2_ concentrations measured by the EC IRGA at the top of the tower (16 or 20 m) and an IRGA (model LI-800, LI-COR Inc.) that sampled air from mid-canopy level (8 m). Storage fluxes were not calculated for the younger site. Half-hourly NEP (–NEE; net ecosystem CO_2_ exchange) was calculated by adding F_c_ and the rate of CO_2_ storage change in the air column below the EC sensors. In the open-path EC system, half-hourly fluxes were derived using 10 min averages that were corrected for the effects of air density fluctuations (WPL correction; Webb et al. 1980). Our closed- and open-path EC systems were compared for about 1 week with a roving closed-path EC system operated by FCRN researchers during a site inter-comparison campaign in 2005. This comparison showed a small difference between our closed-path EC system and FCRN closed-path EC system (Fc__CPEC_ = 0.918 Fc__FCRN_ + 0.11; *R*^2^ = 0.98). The difference was relatively large between our closed-path and open-path EC systems (Fc__OPEC_ = 0.77 Fc__CPEC_−0.6; *R*^2^ = 0.86). Closed-path EC at our 80-year-old site was cross calibrated with AmeriFlux EC system from 8 to 19 July 2019 indicating 3% difference in turbulent fluxes from both EC systems. Fluxes were not adjusted to reconcile the relatively small difference (< 10%) between our open- and closed-path EC systems.

Meteorological variables were measured at the EC sampling heights at all three sites and included: air temperature (Ta) and relative humidity (model HMP45C, CSI; at 28/34, 14 and 2 m heights at the 80-year-old site), wind speed and direction (model 05103-10, R.M. Young Co.), downward and upward photosynthetically active radiation (PAR↓ and PAR↑, model LI-200S, LI-COR Inc.). Soil moisture (model CS-615/616, CSI) was measured at two locations at 5, 10, 20, 50 and 100 cm depths at the 80-year-old site, and at 5, 10, 20, and 50 cm depths at the 45- and 17-year-old sites. Precipitation was measured above the tree height using an all-season, heated, tipping-bucket rain gauge (model 52202, R.M. Young Co.) at the 80-year-old site from 2003 to 2007. Since 2007, precipitation was measured using an accumulation rain gauge (model T200B, Geonor Inc.), a tipping-bucket rain gauge (model TE525, Texas Inst.) in an open area near the 80- and 45-year-old sites, and a heated all-season tipping bucket rain gauge (model 52202, R.M. Young Co.) at the 17-year-old site. Precipitation measurements were cross-checked using data collected at the Environment and Climate Change Canada weather station at Delhi, Ontario. All meteorological and soil data were averaged to half-hour intervals.

### Data processing and gap-filling

Meteorological and CO_2_ flux data were quality controlled following FCRN protocols, described in detail in Brodeur (2014). Data quality was cross checked by AmeriFlux using protocols developed for the Fluxnet2015 data release, when submitting these data to AmeriFlux archives. Gaps in meteorological variables caused by instrument malfunction, power failure, and instrument calibrations and data quality control (1, 4 and 9% at the 80-, 45- and 17-year-old sites, respectively) were filled using estimates from linear regressions with the corresponding data from other sites or from the Environment and Climate Change Canada weather station at Delhi, Ontario (Environment and Climate Change Canada 2020). Remaining erroneous flux data points were removed following Papale et al. (2006). For each half-hourly flux measurement, a ‘flux footprint’ was calculated using a three-dimensional Lagrangian footprint model to retain fluxes within 80% of cumulative flux threshold (Kljun et al. 2003). To remove erroneous flux values during low turbulence periods, a friction velocity (u*) threshold of 0.5 was applied to both daytime and nighttime data, in accordance with previous studies at our sites (e.g., Arain and Restrepo-Coupé 2005; Peichl et al. 2010a). Overall, closed-path EC flux data capture was 87, 90 and 83% at the 80-, 45-, and 17-year-old sites, respectively. However, after footprint and *u*^∗^ threshold filtering, the total portion of NEP data retained was 52, 47 and 17% at the respective sites. These fluxes were well distributed among all seasons over the year, especially while using close-path EC systems. Relatively lower retention of flux data at the 17-year old site was because of limited fetch ranging from northwest to southeast direction and subsequent application of footprint threshold to exclude data when wind was blowing from these directions. Brodeur (2014) has analyzed and further discussed the sensitivity of annual carbon exchanges at our sites due to different lengths of data gaps caused by footprint and *u*^∗^ thresholds.

Daytime RE and gaps in nighttime RE were filled using modelled RE values derived as a function of soil temperature (*T*_*s*_) at 5 cm depth and volumetric water content in the 0–30 cm soil layer (*θ*, estimated utlizing measurements made at 5, 10 and 20 cm depths) using Ordinary Least Squares (OLS) non-linear regression model applied to half-hourly nighttime NEE data (representing RE) as described in Beamesderfer et al. (2020). Half-hourly GEP values were estimated by adding measured NEP to modelled daytime RE. Missing GEP values were modelled using a rectangular hyperbolic function that was fitted to bin-averaged, half-hourly GEP and PAR data (Beamesderfer et al. 2020). Missing nighttime and daytime NEP values were filled using the difference between modelled RE and GEP values. In our study, positive NEP values indicate carbon uptake and negative values indicate carbon loss to the atmosphere. Further details are given in Peichl et al. (2010a), Brodeur (2014), Skubel et al. (2015) and Chan et al. (2018).

### Data analysis

Relative Extractable Water (REW) in the root zone (0–30 cm) was used as a quantitative indicator for dry conditions to assess drought impacts (Black 1979). REW expresses the amount of soil water available for plant-use as a proportion of the maximum possible extractable water for the sites. REW was calculated as

1 $$REW = \frac{{ \theta - \theta_{wp} }}{{\theta_{fc} - \theta_{wp} }}$$

where ${\theta }_{wp}$ is the soil volumetric water content at plant wilting point (0.01 m^3 ^m^−3^) and ${\theta }_{fc}$ is the soil water content at field capacity (0.20 m^3 ^m^−3^), as estimated by McLaren et al. (2008). A dry period was characterized when daily REW was ≤ 0.4 and a drought year was categorized when REW was ≤ 0.4 for more than 2 months (62 days) each year. Several studies in literature have used a REW threshold of 0.4 to define water stress or drought conditions in forest ecosystems (Bréda et al. 1995; Granier et al. 2007; Davi et al. 2006; Maseyk et al. 2008). The “hot days” were defined as days when daily maximum temperature (T_max_) ≥ 27.5 °C, which is the 90th percentile of daily Tmax over the 30-year reference period (1971–2000) in the region prior to the start of our study (Environment and Climate Change Canada weather station at Delhi, Ontario). The “hot years” were categorized as the years that had 30 or more hot days in a given year. While quantitative and climatic sensitivity analysis was conducted and reported using daily values, annual characterization of hot and dry years was only used for discussion purposes. Considering the climatic seasonality in the region, the seasons were defined by calendar months with spring consisting of April and May, summer consisting of June, July, August, and September, autumn consisting of October and November, and winter consisting of December, January, February and March.

The impacts of extreme weather events may potentially be masked by the inter-annual and seasonal weather variations, and differences in forest growth among our different-age sites. Therefore, time series of carbon fluxes (GEP, RE and NEP) and the environmental variables (Ta and REW) were detrended and normalized over the study period, following Xu et al. (2020). The slope of linear relationships between daily carbon flux and environmental variables indicated the sensitivity of carbon fluxes to climate anomalies, demonstrating the direct effect of climate constraints on the carbon balance (Schwalm et al. 2010; Wu and Chen 2013). In the monthly data, negative correlation with Ta anomalies indicated heat stress, while positive correlation with REW anomalies indicated drought stress. The daily anomalies were pooled together over bi-weekly periods to calculate the sensitivity indices (Xu et al. 2020). At the daily level, the slopes of REW and carbon flux anomalies were multiplied by − 1. Therefore, negative values of both Ta and REW sensitivity indicate that heat and drought stress significantly decreased carbon fluxes. The age effect on sensitivity to heat and drought stresses were evaluated by comparing the sensitivity indices of three sites.

A residual analysis was also conducted using daily GEP, RE, and NEP from the closed-path EC systems at each site. The correction of Ta models’ residuals of GEP, RE, and NEP with REW was tested for all the data, as well as for hot days (T_max_ ≥ 27.5 °C). All the calculations and analysis were conducted in MATLAB software (The Mathworks Inc.).

## Results

### Climate

**Fig. 1:**
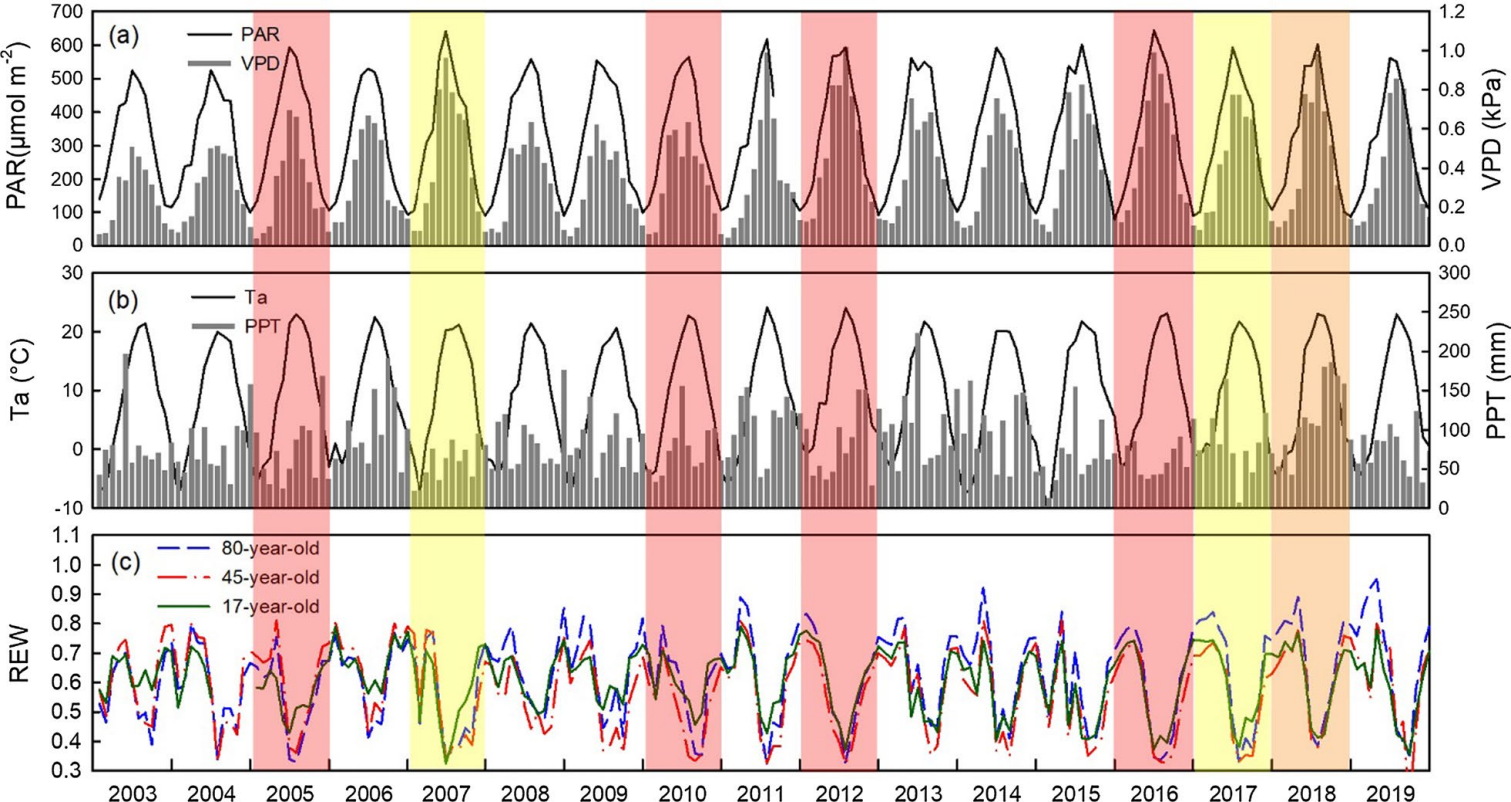
Monthly values of **a** photosynthetically active radiation (PAR) and vapor pressure deficit (VPD), **b** mean air temperature (Ta) and total precipitation (PPT), and **c** soil Relative Extractable Water (REW) in the 0–30 cm soil layer at each site. Dry years are shaded as yellow (2007, 2017), hot year is shaded as orange (2018), and concurrent hot and dry years are shaded as red (2005, 2010, 2012 and 2016)

The climate over the study period (2003 to 2019) was characterized by cold winters and hot and humid summers (Fig. 1). This region experienced record or near-record summer heat events in 2005, 2010, 2012, 2016 and 2018 (Shein 2006; Phillips 2006, 2011, 2013, 2017, 2019). Observed absolute maximum Ta (half-hourly values) at our sites ranged from 34.48 °C in 2012 to 28.70 °C in 2004, with a mean ± standard deviation value of 31.50 ± 1.36 °C from 2003 to 2019. The mean summer (June through September) Ta were 21.3, 20.7, 20.9, 20.9 and 21.0 °C in 2005, 2010, 2012, 2016 and 2018, respectively, which were higher than the 17-year mean summer Ta of 19.9 °C.

High soil water content, as illustrated by REW, was generally observed in late winter and early spring, with peak REW values occurring in March–April due to snowmelt and/or spring rainfall events (Fig. 1). A rapid decline in REW often occured in May, coinciding with an increase in photosynthetic activity and evapotranspiration. Overall, the seasonal dynamics of REW were consistent among the three sites, with the 45-year-old stand generally having the lowest REW values in the summer.

The sites experienced dry conditions in 2005, 2007, 2010, 2012, 2016 and 2017, where monthly mean REW values dropped below 0.40 in the late summer. Therefore, 2007 and 2017 were characterized as years that experienced drought events, 2018 experienced heat events and 2005, 2010, 2012, and 2016 experienced concurrent heat and drought events. Year 2018 had the second warmest summer temperatures which were followed by high precipitation in the late growing season and onwards (Fig. 1b).

### Age-related dynamics of carbon fluxes

**Fig. 2:**
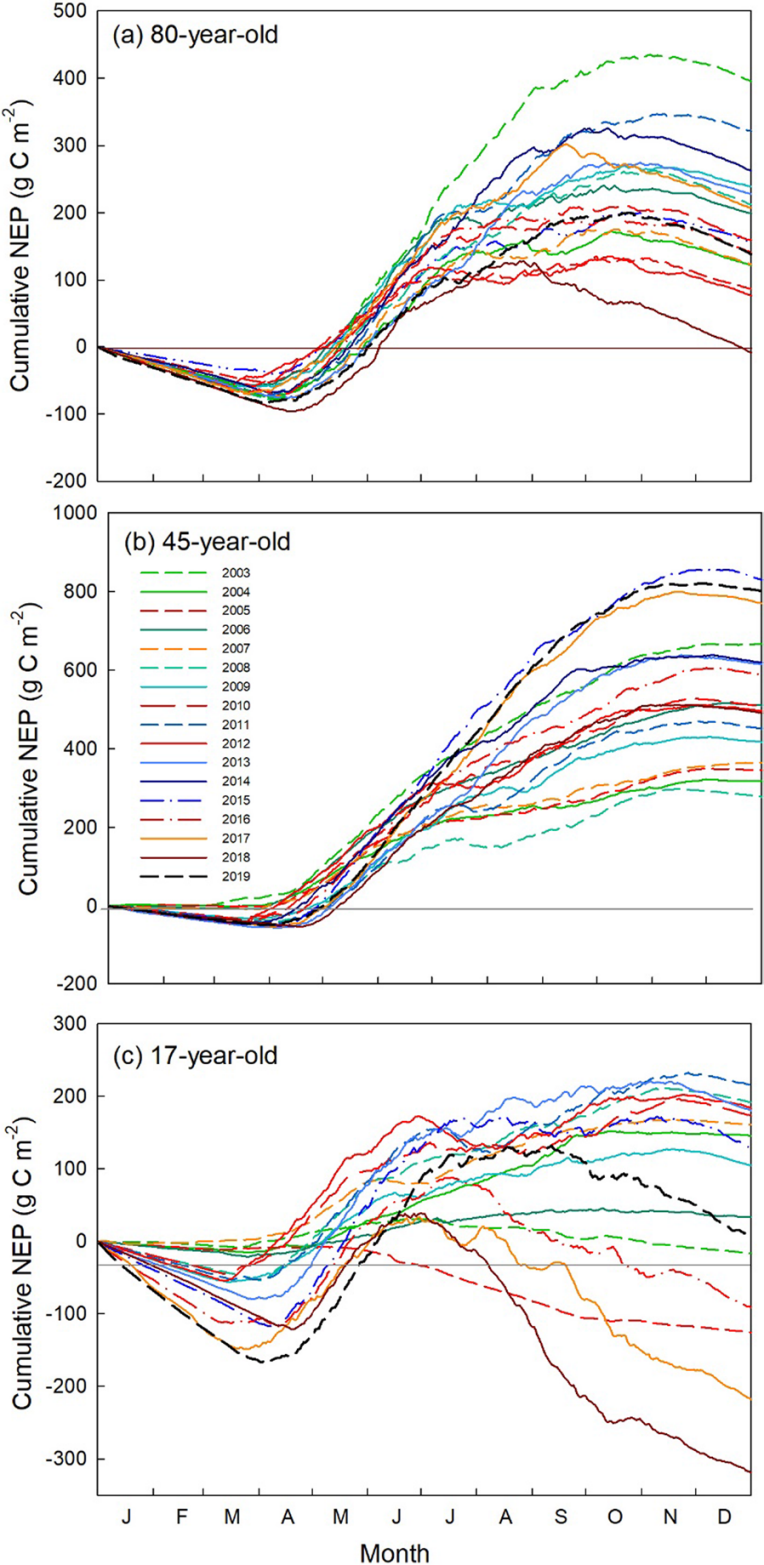
Cumulative annual net ecosystem productivity (NEP) at the **a** 80-year-old, **b** 45-year-old, and **c** 17-year-old forests. Dry years are shown as yellow (2007, 2017), hot year as burgundy (2018), and concurrent hot and dry years as red (2005, 2010, 2012 and 2016) shades of coloured lines

The seasonal course of NEP at all three forest sites is shown in Fig. 2. In the 80-year-old stand, the lowest NEP values were observed in the growing seasons of 2005, 2012 and 2018, when the forest experienced concurrent heat and drought stresses. These stress events turned the forest into a small carbon source on annual basis. In contrast, 2015, which experienced a mild drought in the early and late growing season, but a large precipitation event (> 150 mm, Fig. 1c) in June, was the most productive year of the study period, with no usual mid-summer decline in NEP (Fig. 2). In the 45-year-old stand, 2005 (hot and dry), 2008 (cold, low VPD) and 2007 (dry) had much smaller summer NEP values compared to other years. The 17-year-old stand experienced a large decrease in NEP after May–June in 2005 and 2016 (hot and dry), 2017 (dry) and 2018 (hot) that caused the stand to become a source of carbon. In general, years with concurrent drought and heat events had lower cummulative NEP values at all sites as explained in the following section.

**Fig. 3:**
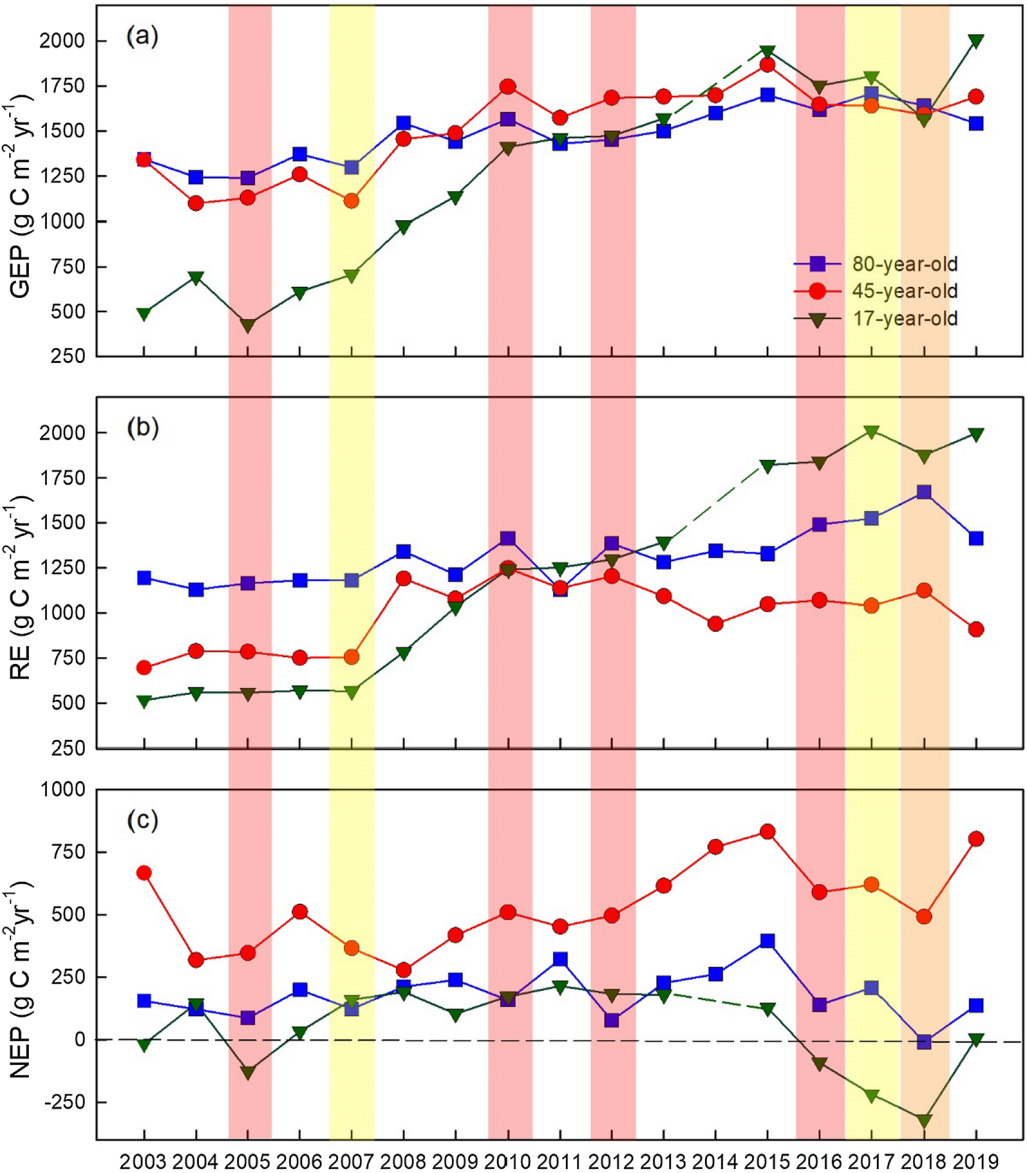
Annual total **a** gross ecosystem productivity (GEP), **b** ecosystem respiration (RE), and **c** net ecosystem productivity (NEP) in the 80-, 45-, and 17-year-old forests. Dry years are shaded as yellow (2007, 2017), hot year is shaded as orange (2018), and concurrent hot and dry years are shaded as red (2005, 2010, 2012 and 2016)

In the 80-year-old stand, annual NEP showed a consistent decline from 2006 to 2018, recording the lowest NEP in 2018 when the forest became a net carbon source (− 9 g C m^−2^ yr^–1^). In the 45-year-old stand, annual NEP values showed a consistent increase from 2008 to 2015, with the highest NEP of 831 g C m^–2^ yr^–1^ in 2015, which was a wet year, followed by 807 g C m^–2^ yr^–1^ in 2019. The 17-year-old stand showed a mixed pattern of annual NEP values during the initial few years, where the site was a source of carbon in two out of the four years. The stand became a consistent carbon sink (NEP > 0) after 5 years of its plantation until 2015. After this, the stand became a source of carbon from 2006 to 2018, with the lowest NEP value of − 319 g C m^–2^ yr^–1^ observed in 2018. It was remarkable to observe a very similar trend of NEP decline in all three stands over three consecutive years experiencing extreme events: 2016 (hot and dry), 2017 (dry), and 2018 (hot) (Fig. 3c).

Overall, the mean annual NEP ± standard deviation over the 17-year study period was 180 ± 96, 538 ± 168 and 64 ± 165 g C m^–2^ yr^–1^ in the 80-, 45- and 17-year-old forests, respectively (Table 2). Carbon sequestration rates and forest productivity were highest in the 45-year-old stand (Figs. 2 and 3). Changes in NEP across all stand ages showed that the 45-year-old and 17-year-old forests had large inter-annual variations in annual NEP, while the 80-year-old forest had smaller and less variable NEP.

**Table 2:** Annual values of gross ecosystem productivity (GEP), ecosystem respiration (RE) and net ecosystem productivity (NEP) in the 80-year-old, 45-year-old, and 17-year-old forest sites from 2003–2019

Year	80-year-old			45-year-old			17-year-old			Ta	*PPT*
	GEP	RE	NEP	GEP	RE	NEP	GEP	RE	NEP		
2003	1344	1194	155	1340	695	666	494	515	− 17	8.17	913
2004	1244	1129	123	1098	788	318	695	558	145	8.54	955
2005	1239	1163	87	1129	785	346	429	557	− 126	9.11	863
2006	1372	1181	199	1258	750	511	611	569	34	9.82	1478
2007	1298	1180	123	1111	755	366	706	566	160	9.23	712
2008	1545	1342	212	1456	1189	279	976	784	191	8.58	1021
2009	1443	1212	239	1488	1078	418	1137	1034	105	8.39	995
2010	1567	1415	160	1746	1247	509	1412	1241	173	9.54	896
2011	1430	1128	322	1574	1136	452	1462	1252	216	9.56	1293
2012	1452	1385	77	1685	1203	496	1473	1297	183	11.16	1001
2013	1501	1282	228	1691	1091	615	1571	1394	180	9.09	1266
2014	1601	1345	263	1698	939	771	NA	NA	NA	8.08	1188
2015	1701	1328	395	1869	1048	831	1950	1823	128	9.48	811
2016	1617	1491	141	1648	1070	589	1753	1841	− 91	10.03	778
2017	1709	1525	208	1641	1039	620	1805	2014	− 218	9.92	986
2018	1641	1670	− 9	1591	1123	549	1569	1877	− 319	9.42	922
2019	1543	1415	138	1695	916	807	2008	1998	6	8.96	911
Mean	1485	1317	180	1513	979	538	1253	1208	64	9.24	999

NA—Carbon fluxes were not reported at the 17-year-old site in 2014 due to missing data caused by an IRGA malfunction that affected flux data quality

Units for all carbon fluxes are g C m^−2^ yr^−1^. Annual mean air temperature, Ta (°C) and annual total precipitation, PPT (mm yr^−1^) are also given

In terms of GEP, we observed a gradual increase in annual GEP in the 80- and 45-year-old stands and a more pronounced increase in the 17-year-old stand, where it increased from 494 g C m^–2^ yr^–1^ in 2003 to 1998 g C m^–2^ yr^–1^ in 2019 (Fig. 3, Table 2). After 2010 (hot and dry), all three stands showed a very similar trend in increasing annual GEP (Fig. 3). However, in 2019, annual GEP of the 17-year stand exceeded the two older stands. A substantial increase in RE was observed in the 80-year-old stand from 2016 to 2018, with the highest RE (1670 g C m^−2^ yr^–1^) observed in 2018 (hot), when the stand became a small carbon source. The annual RE in the 45-year-old stand increased from 2007 to 2010 and then leveled-off from 2011 to 2018, while annual RE in the 17-year-old stand consistently increased from 2007 to 2019 (Fig. 3).

### Sensitivity of carbon flux anomalies to and heat and drought stresses

**Fig. 4:**
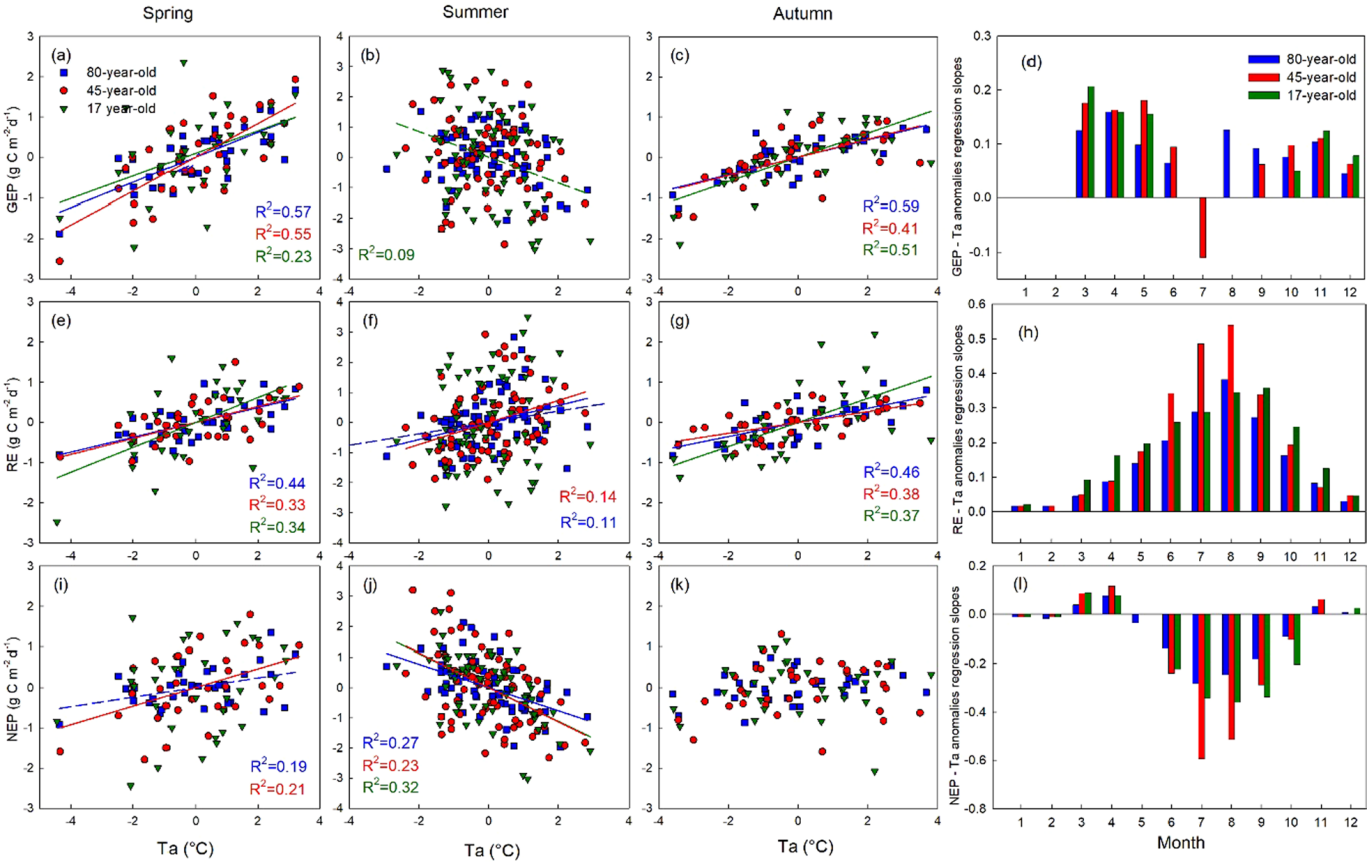
Correlations between monthly air temperature (Ta) anomalies and **a**–**c** monthly gross ecosystem productivity (GEP), **e**–**g** ecosystem respiration (RE), and **i**–**k** net ecosystem productivity (NEP) anomalies in spring (AM), summer (JJAS) and autumn (ON) in the 80-, 45-, and 17-year-old stands. Significant linear regressions at *p* < 0.01 are shown as solid lines. Significant linear regressions at 0.01 < *p* < 0.05 are shown as dash lines. The slope of the linear relationship between daily Ta anomalies and daily GEP (**d**), RE (**h**), and NEP (**l**) in each month are shown when the regression was significant at *p* < 0.05

**Fig. 5:**
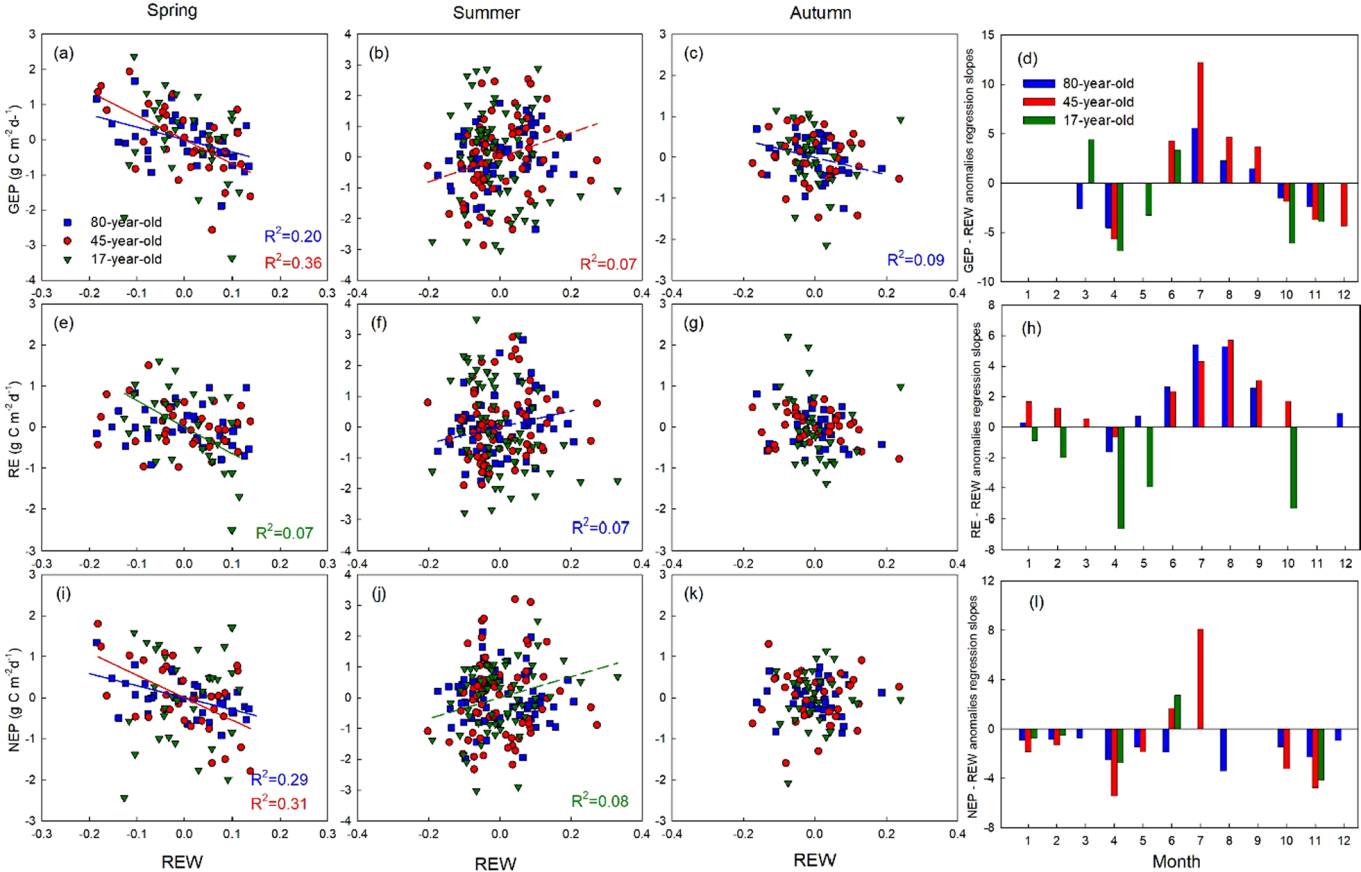
Correlations between monthly relative extractable water (REW) anomalies and monthly gross ecosystem productivity (GEP) (**a**–**c**), ecosystem respiration (RE) (**e**–**g**), and net ecosystem productivity (NEP) (**i**–**k**) anomalies in spring (AM), summer (JJAS) and autumn (ON) in the 80-, 45-, and 17-year-old stands. Significant linear regressions at *p* < 0.01 are shown as solid lines. Significant linear regressions at 0.01 < *p* < 0.05 are shown as dashed lines. The slope of the linear relationship between daily REW anomalies and daily GEP (**d**), RE (**h**) and NEP (**l**) in each month are shown when the regression was significant at *p* < 0.05

At all three sites, the anomalies of mean monthly air temperature (Ta) were positively and significantly correlated with GEP and RE anomalies in spring and autumn (Fig. 4a, c, e and g), while no correlation was found with NEP anomalies during these seasons, except in the 45-year-old forest in spring (Fig. 4i and k). In the summer, GEP anomalies were negatively correlated with Ta anomalies in the 17-year-old and 45-year-old stands, indicating less carbon uptake during warm periods (Fig. 4b, f), while positive correlations were found between RE and Ta anomalies in the 80-year-old and 45-year-old stands, suggesting higher carbon loss with increasing temperatures. The NEP anomalies were significantly, but negatively, correlated with Ta anomalies in summer with *R*^2^ of 0.31, 0.32 and 0.47 for the 80-, 45- and 17-year-old stands, respectively, indicating less net carbon uptake under heat stress (Fig. 4j). The impact of REW anomalies on GEP, RE, and NEP anomalies was not as strong as the impact of Ta anomalies (Fig. 5). However, NEP anomalies were slightly negatively correlated with REW anomalies in most of the months and stands (Fig. 5l). Positive correlations, which indicated drought stress, were only observed in June and July in the 45-year-old stand, and in June in the 17-year-old stand (Fig. 5l).

**Fig. 6:**
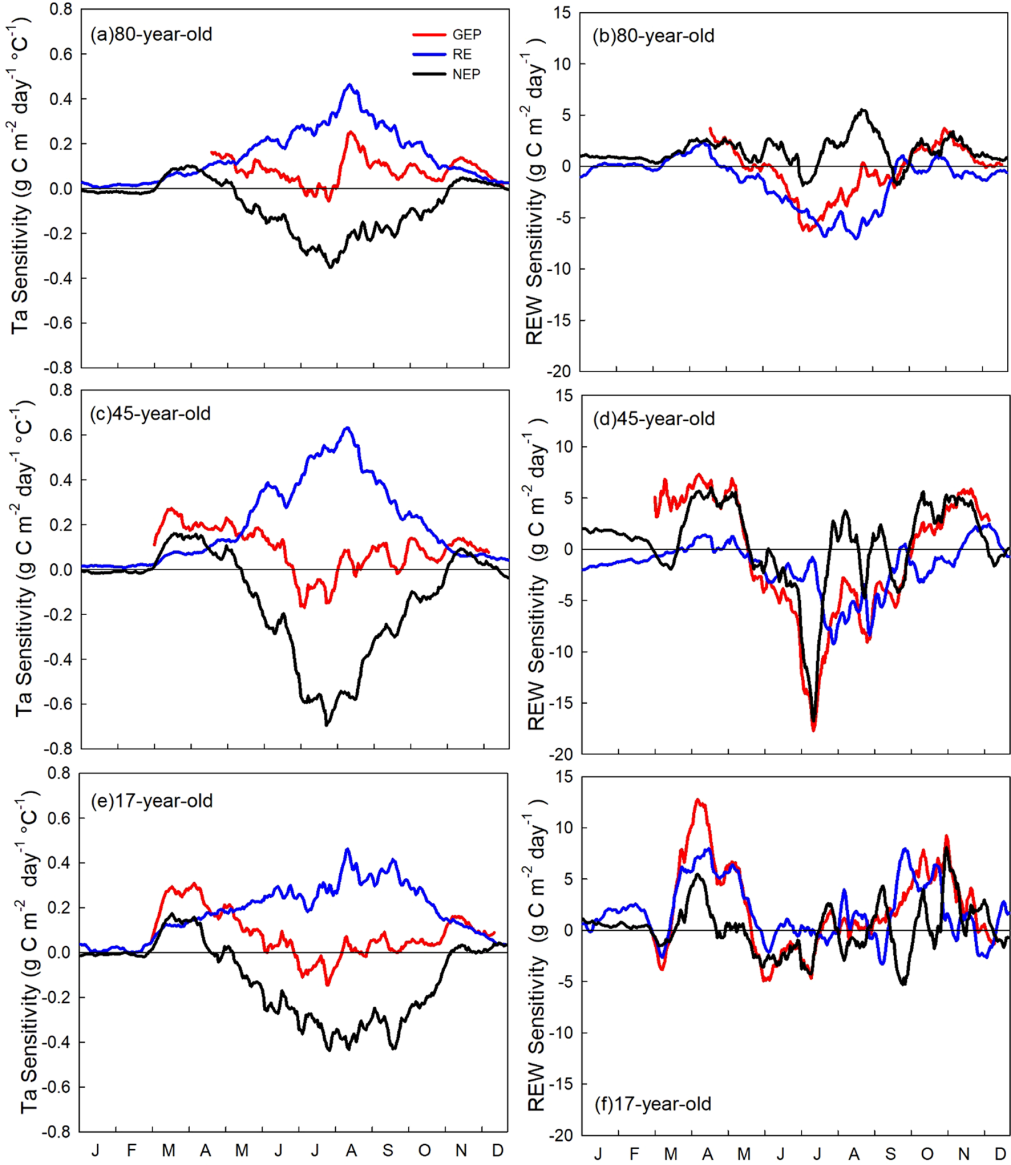
Daily sensitivities of carbon fluxes to air temperature (Ta) and dryness represented by relative extractable water (REW) anomalies. Curves show the mean sensitivity in the 15 day moving windows. Data were detrended and normalized to remove forest growth and long-term climate variability impacts. When the flux vs climate variable correlation for 15 day moving averages was significant (*p* < 0.05), the slope of the regression showed the effect of climate constraints on the carbon fluxes (following Schwalm et al. 2010; Wu and Chen 2013)

The sensitivities of daily GEP, RE, and NEP anomalies to Ta and REW anomalies at all three stands are shown in Fig. 6. Here, negative sensitivity values represent heat (dNEP/dTa < 0) and drought (dNEP/dREW < 0) stress. In early summer (June), all three stands showed decreases in GEP with Ta anomalies (dGEP/dTa < 0), while RE increased with Ta anomalies (dRE/dTa > 0) (Fig. 6c). Therefore, the sensitivity of NEP to Ta anomalies was negative in the summer at all three stands (Fig. 6e). The average summer NEP sensitivity to Ta anomalies was approximately 0.21, 0.36, 0.23 g C m^−2^ day^−1^ °C^−1^, for the 80-, 45- and 17-year-old stands, respectively, suggesting a strong heat stress impact in the summer. The sensitivity of NEP to Ta anomalies was the largest at the 45-year-old stand and smallest at the 80-year-old stand (Fig. 6d, h, l).

The drought stress (REW) showed a persistent positive effect on both GEP and RE in the two younger stands in the spring. In the summer, dry conditions had a similar negative effect on GEP and RE in the 80-year-old stand (dGEP/dREW < 0, Fig. 6b); therefore, NEP was not sensitive to REW anomalies. The 45-year-old stand showed a strong drought stress impact on NEP, which was mostly driven by the decreases in GEP (dGEP/dREW < 0, Fig. 6d). The sensitivity of GEP anomalies to REW anomalies gradually increased from negative to positive values during the summer in the 17-year-old stand and the drought stress had less of an impact on RE anomalies than GEP anomalies. As a result, the REW anomalies had a small negative effect on NEP anomalies in the early summer (−dNEP/dREW < 0, Fig. 6f), but the effect was reduced in the late summer in the youngest stand.

**Fig. 7:**
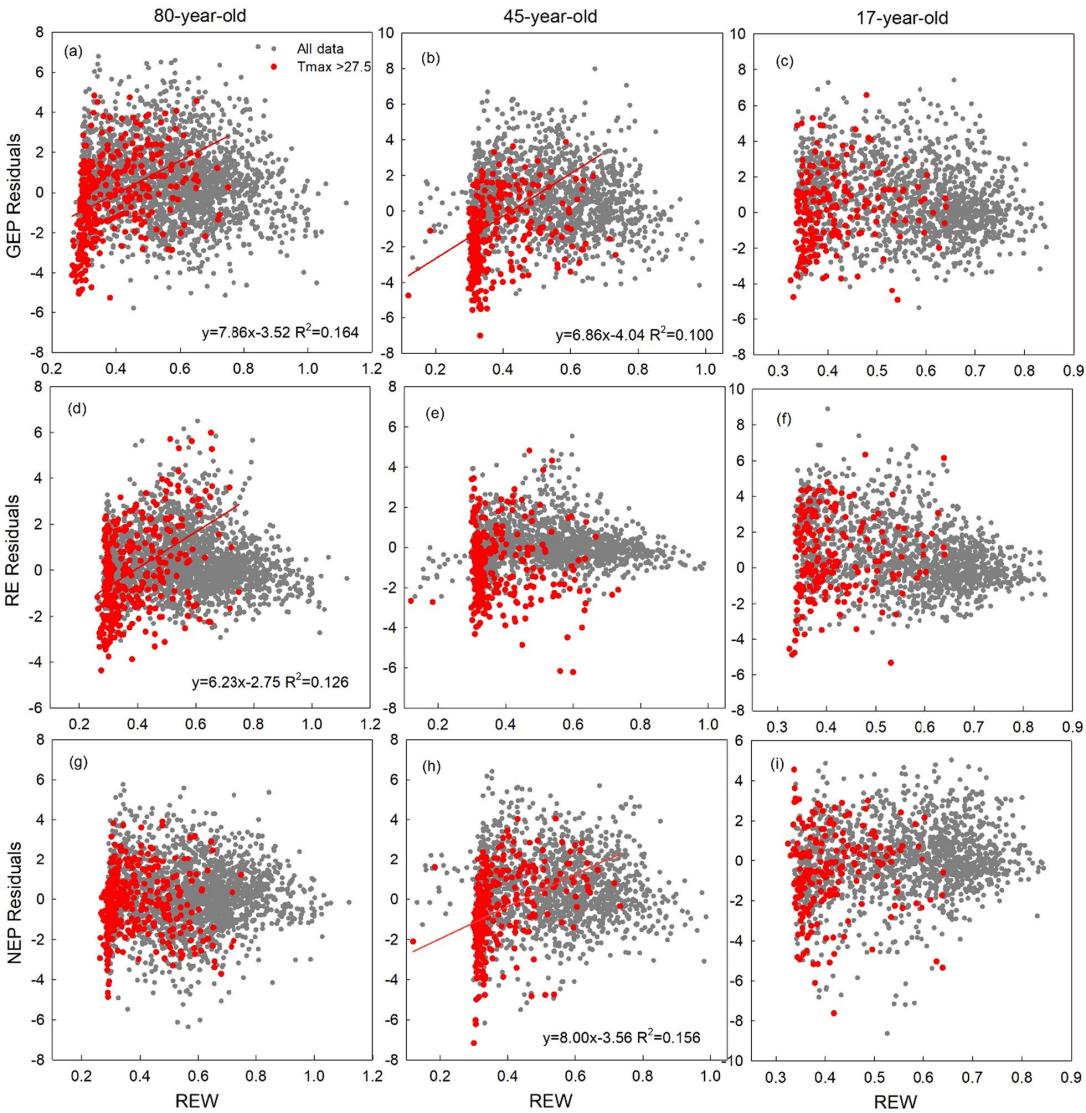
Correlations between daily gross ecosystem productivity (GEP), ecosystem respiration (RE), and net ecosystem productivity (NEP) residuals of REW using data from all days with no precipitation (gray dots) and using data from only hot days (red dots) with no precipitation. Hot days were defined as those when daily maximum air temperature, T_max_ ≥ 27.5 °C. Significant linear regressions at *p* < 0.01 are shown as solid lines

Residuals of GEP, RE, and NEP, in their Ta-model, were not significantly correlated with REW at all three sites (gray dots in Fig. 7). However, when data from only hot days (T_max_ ≥ 27.5 °C) were analyzed, the residuals of GEP were negatively correlated with REW in the 80- and 45-year-old stands (Fig. 7a, b). Residuals of RE and NEP were negatively correlated with REW at the 80- and 45-year-old stands, respectively (Fig. 7d, h).

## Discussion

### Age-related dynamic of carbon fluxes

Among all three different age forests, maximum net carbon uptake occurred in the 45-year-old stand, while the 80-year-old and 17-year-old stands were moderate to small carbon sinks (Table 2; Fig. 3). The 17-year-old stand became a consistent net carbon sink after the fifth year of its establishment (Chan et al. 2018). Clark et al. (2004) also showed similar results in their study in a chronosequence of slash pine plantations in Florida, USA. Achieving early carbon sink status in our young plantation forest was in contrast to naturally regenerating stands that may take many years to become a net carbon sink after harvest or natural disturbance because of the decomposition of large amounts of soil organic matter present at these sites (Kowalski et al. 2003; Humphreys et al. 2006; Amiro et al. 2010; Baldocchi 2019).

Overall, the 80-year-old stand showed smaller inter-annual variability in NEP as compared to younger stands, specifically until 2012. A selective thinning conducted in the 80-year stand in 2012 caused a substantial reduction in canopy cover (33%) and basal area (13%). In addition, this stand experienced hot and dry conditions in the summer causing a substantial decrease in net carbon uptake in 2012, with an annual NEP value of 77 g C m^−2^ yr^−1^, compared to the mean annual NEP of 180 ± 96 g C m^−2^ yr^−1^ over the study period. Decreases in NEP were largely due to increases in RE, while there was a small reduction in GEP when compared to the mean annual GEP over the study period. A number of factors may have caused the increase in RE in 2012, including warmer temperatures, more organic matter on the ground, decomposition of fine roots after thinning and disturbance of soil organic matter due to the machine-harvesting process, although care was taken during harvest to avoid major soil disturbance. At the 80-year site, Skubel et al. (2015) found a slight increase in transpiration in remaining trees, despite 2012 being a warm and dry year. Past studies have reported that thinning results in greater availability of soil water to remaining trees, leading to higher transpiration and hence photosynthesis (Bréda et al. 1995; Reid et al. 2006; Chen et al. 2020a). This may have been the case in our 80-year-old stand. However, the relatively small decline in GEP, despite the removal of 1/3 of trees at our site in 2012, might also be explained by the increase in photosynthetic activity of the understory, which was exposed to more sunlight after harvesting due to a reduction in overstory canopy. Similar observations have been made by other studies, where thinning did not cause a large decline in productivity due to compensatory carbon uptake by the understory vegetation (Campbell et al. 2009). Hence, we suggest that the lower NEP observed in 2012 was due to the combined effects of thinning, and heat and drought stress.

### Sensitivity of carbon fluxes to heat stress

Our sensitivity analysis for heat stress indicated that all three different-aged stands responded to temperature anomalies differently over the course of the year. In the spring and autumn, most of the metabolic processes were limited by energy when the air and soil temperatures were relatively low. Therefore, positive temperature anomalies generally increase photosynthesis and ecosystem respiration. Since GEP and RE responded to temperature in a similar way, overall higher temperatures did not have a significant impact on the NEP. In the summer, GEP was likely constrained directly by thermal damage to the photosynthetic system and indirectly due to reduced stomatal conductance and leaf water potential under heat stress (Rennenberg et al. 2006; Williams et al. 2014; Ruehr et al. 2016; Baldocchi 2019). Potentially, RE can also be limited by high temperature as a result of carbon starvation (autotrophic respiration) or reduction in soil and litter respiration due to their sensitivity to temperature and water availability (heterotrophic respiration) (McDowell et al. 2008). However, the range of temperature during heat events did not surpass the optimal temperature of respiration at our sites; therefore, RE increased with increasing temperature in our study (Duffy et al. 2021). Consequently, NEP (which is the difference of GEP and RE) was strongly, but negatively correlated with Ta in all three stands (Fig. 5h). Because of these contrasting responses of GEP and RE, summer temperature was the most important determinant of annual NEP at our forest sites. Our results agree with von Buttlar et al. (2018), who found that heat without dryness increased RE but did not have much impact on GEP leading to overall reduction in NEP.

### Sensitivity of carbon fluxes to drought stress

Our sensitivity analysis for drought stress indicated that the effects of drought (as indicated by low REW values) were highly dependent on the water demand of the stand during different seasons. During spring, soil water content, and hence REW, was relatively high following snowmelt. Autumn was also relatively wet because of lower atmospheric water demand, reduced transpiration, and larger rainfall events. Overall, drought did not have a strong impact on carbon fluxes in spring and autumn in these different-age forests. However, dry spring conditions led to higher GEP and NEP in the 80-year-old and 45-year-old stands (Fig. 5a, i). It may be caused by stimulated growth due to warm temperature in dry years (concurrent events), where photosynthesis would have benefited due to less cloudy conditions and higher PAR in early spring (Suseela and Dukes 2013). Our stands are evergreen forests and are able to start photosynthesizing immediately in spring once conditions are right, unlike deciduous trees that require leaf-out and development to occur first.

In summer, both GEP and RE were suppressed by drought stress in the 80-year-old stand. A multi-site inter-annual analysis (Doughty et al. 2015) and a warming and precipitation-controlled experiment (Suseela and Dukes 2013), illustrated that drought can reduce autotrophic respiration, which is a major component of RE at our sites (Peichl et al. 2010b). However, some other studies have shown that drought could also impact allocation of carbon to various pools, where more carbon may be allocated to roots to access water from deeper soil, reducing the impacts of drought on photosynthetic uptake and avoiding post-drought carbon starvation or mortality (Peng et al. 2011; Doughty et al. 2015). Our results support these studies and suggested that summer droughts reduced GEP and RE in a similar way, while NEP was not significantly impacted by drought stress alone. von Buttlar et al. (2018) reported similar findings for drought stress in the absence of heat in their synthesis study. However, the sensitivity of our forests to dryness decreased for droughts occurring in late summer, which is a common phenomenon in the region (Hanson and Weltzin 2000). It indicates long-term adaptation of trees to drought stress.

### Sensitivity of carbon fluxes to concurrent heat and drought stresses

Our study illustrated strong impact of concurrent heat and drought stresses on forest carbon cycle. We found that while our different-aged forests were sensitive to temperature variation, their sensitivity to dryness was highly dependent on the timing of the drought and temperature stress as shown in the past experimental (Duan et al. 2016), synthesis (von Buttlar et al. 2018) and modeling (Zscheischler et al. 2014) studies, suggesting that forests undergo a greater degree of stress and NEP reduction under concurrent heat and drought events than under individual heat and drought events.

Our analysis further showed that the residuals of temperature-modeled carbon fluxes were not correlated with REW at all the sites. However, when the residuals analysis was conducted using data for hot days (T_max_ > 27.5° C) only, REW became a significant driver of GEP and RE. This result supported the findings of Williams et al. (2014) who found that water stress impacts on forest carbon fluxes start above a certain temperature threshold. It is likely that, when the temperature is relatively low, photosynthesis, respiration, and transpiration are limited by temperature. Consequently, a decrease in soil water may not have a significant impact on carbon fluxes. However, when the temperature is high and the soil water storage cannot provide enough water to maintain higher rates of transpiration, decreases in canopy conductance may lead to a decline in GEP, and hence NEP (Duarte et al. 2016). Our results confirmed the interaction between heat and drought events and validated the hypothesis that drought stress has a much more significant impact on NEP when it occurs concurrently with heat stress (Sippel et al. 2016; von Buttlar et al. 2018).

Our results showed that forest response to heat and drought stress differs among different stand ages. In our three age-sequence sites, the middle aged forest (45 years) was more sensitive to both heat and drought stress than the younger and older forests. Young forests such as our 17-year-old stand have shallower roots than older stands (Peichl and Arain 2006; Peichl et al. 2010a; Chan et al. 2018) and they predominantly rely on water content in the upper soil layers. Therefore, depletion of water from upper soil layers during high heat periods may potentially have a greater adverse impact on their carbon fluxes. However, younger forest has smaller biomass and photosynthetic capacity (GEP), resulting in relatively lower water demand during the growing season. Therefore, net ecosystem productivity of younger forests is not much adversely impacted, and they are less sensitive to heat and drought extremes as compared to middle aged stands. Older forests, such as our 80-year-old stand, with a well-established and deeper root system, are better buffered from seasonal droughts (Wu et al. 2017). Our 80-year-old stand conserved water when soil water availability was low, causing a negative feedback through the trade-off between carbon assimilation and transpiration under drier conditions. This partly explains why the NEP in the 80-year-old stand was not limited by REW (Fig. 5l) and was less sensitive to abnormally high temperatures in summer (Fig. 4j, l). Our findings agree with other studies (e.g., Irvine et al. 2002; Yang et al. 2010; Gao et al. 2017; Chen et al. 2020b; Duffy et al. 2021) and further imply that mature forests are more resilient to drought stresses. It is also likely that long-term forest management activities, such as selective thinning, may have increased the forest’s resilience to drought by reducing stand density (Giuggiola et al. 2013) or by introducing more drought resistant secondary deciduous species (Arthur and Dech 2016). Our results imply that the more frequent climate extremes in the future may have a profound impact on forest carbon sink in managed temperate forests, as the most productive middle aged forest are the most sensitive to heat and drought stresses.

### Response of carbon fluxes to consecutive multiple-year extreme events

Our sites experienced simultaneous heat and drought events in 2016, followed by severe drought in 2017 and a summer heatwave in 2018. Consequently, all three forests had a significant and consistent decrease in annual NEP from 2016 to 2018, where by as of 2018, NEP had declined by 447, 283 and 404 g C m^−2^ at the 80-, 45-, and 17-year-old forest, respectively, as compared to annual NEP recorded in 2015. The youngest stand became a net source of carbon for all three of these years and the oldest stand became a small source of carbon for the first time in 2018, since observations started in 2003. All three sites showed a very similar pattern of this NEP decline over this three-year period (Fig. 3c). This indicates that our hypothesis suggesting that the NEP of younger stands will be much more impacted by consecutive occurrence of extreme events was not validated. While the decline in NEP at the two younger stands was caused by decreases in GEP and simultaneous increases in RE, the decline in NEP at the 80-year-old stand was primarily caused by the increase in annual RE from 2016 to 2018. Therefore, although the GEP in the oldest stand was resilient to the consecutive extreme events, RE kept on increasing, likely due to larger stand biomass and soil organic pool compared to younger stands, causing reduction in NEP.

## Conclusions

This study explored the response of carbon fluxes to heat and drought stresses in three different-aged temperate conifer forests from 2003 to 2019. Our results illustrated that when heat and drought events occurred either simultaneously during the early growing season or concurrently over multiple years, they had a significant negative impact on annual NEP in all three forests. When all data were considered, Ta was the dominant control of carbon fluxes at all three sites; however, when the analysis was conducted using data for hot days only (T_max_ > 27.5 °C), REW became a significant driver of GEP and RE. Our results also showed that declines in NEP of the younger stands was similar to that of the older stand in years with consecutive occurrence of extreme events (2016–2018), although the underlying drivers of that trend varied. While the decline in NEP at the two younger stands was caused by decreases in GEP and simultaneous increases in RE, the decline in NEP at the 80-year-old stand was primarily caused by the increase in annual RE, due to a larger stand biomass and soil organic carbon pool compared to younger stands and small change in GEP. Plantation forests in Eastern North America are managed with the vision to enhance their growth, resilience to stresses, and to maximize their carbon sequestration potential (Meehl et al. 2007; Sippel et al. 2016). As heat and drought events are likely to be more frequent in the area, due to the predicted warmer climate in the future (IPCC 2014; 2018; Niinemets 2010; Xu et al. 2020; Chen et al. 2020a; Fernández-Martínez et al. 2020), their combined effects especially over multiple years, may have serious implications for net carbon sequestration in temperate conifer forests of Eastern North America. The impact of concurrent and consecutive extreme events should be considered while developing forest management practices for climate resiliency and sustainability with the aim of climate change mitigation by way of greenhouse gas reduction. Well managed and sustainable forests will have better capabilities to cope with the risks and impacts caused by extreme events. Our results and long-term flux, meteorological and biometric data in three different-aged forests will also help in improving carbon exchange processes in terrestrial ecosystem models.

## Data Availability

The data sets used during this study are available at the AmeriFlux website (DOIs given in the methods section) or from the corresponding author upon request.

## Abbreviations

CO_2_: Carbon dioxide
EC: Eddy covariance
GEP: Gross ecosystem productivity
NEP: Net ecosystem productivity
PAR: Photosynthetically active radiation
RE: Ecosystem respiration
REW: Relative extractable water
Ta: Air temperature
T_max_: Maximum air temperature
Ts: Soil temperature
*u**: Friction velocity
VPD: Vapour pressure deficit
